# Mixed Methods Process Evaluation of Behavioral Support and Nicotine Replacement Therapy for Smokeless Tobacco Cessation in Bangladesh, India, and Pakistan

**DOI:** 10.1093/ntr/ntag004

**Published:** 2026-01-08

**Authors:** Faraz Siddiqui, Fayaz Ahmad, Fariza Fieroze, Silwa Lina, Laraib Mazhar, Anila Mahmood Nawaz, Varsha Pandey, Prashant Kumar Singh, Narjis Rizvi, Shikha Saxena, Ray Croucher, Ian Kellar, Heather Thomson, Javaid Ahmed Khan, Rumana Huque, Romaina Iqbal, Kamran Siddiqi, Mona Kanaan, Linda Bauld, Cath Jackson

**Affiliations:** Department of Health Sciences, University of York, York, UK; Institute of Public Health and Social Sciences, Khyber Medical University, Peshawar, Pakistan; ARK Foundation, Dhaka, Bangladesh; ARK Foundation, Dhaka, Bangladesh; Department of Community Health Sciences, Aga Khan University, Karachi, Pakistan; Department of Community Health Sciences, Aga Khan University, Karachi, Pakistan; Division of Preventive Oncology & Population Health, Indian Council of Medical Research-National Institute of Cancer Prevention and Research (ICMR-NICPR), Noida, India; Division of Preventive Oncology & Population Health, Indian Council of Medical Research-National Institute of Cancer Prevention and Research (ICMR-NICPR), Noida, India; Department of Community Health Sciences, Aga Khan University, Karachi, Pakistan; Division of Preventive Oncology & Population Health, Indian Council of Medical Research-National Institute of Cancer Prevention and Research (ICMR-NICPR), Noida, India; School of Health Sciences and Technology, UPES, Dehradun 248007, Uttarakhand, India; Department of Health Sciences, University of York, York, UK; School of Psychology, Interdisciplinary Centre of the Social Sciences, Sheffield, UK; Leeds City Council, Leeds, UK; Section of Pulmonary and Critical Care Medicine, Department of Medicine, Aga Khan University Hospital, Karachi, Pakistan; ARK Foundation, Dhaka, Bangladesh; Department of Community Health Sciences, Aga Khan University, Karachi, Pakistan; Hull York Medical School, University of York, York, UK; Department of Health Sciences, University of York, York, UK; Usher Institute and Behavioural Research UK, University of Edinburgh, Edinburgh, UK; Department of Health Sciences, University of York, York, UK

## Abstract

**Introduction:**

Interventions for quitting smokeless tobacco are lacking in South Asia. In a pilot trial, we explored the feasibility of delivering and evaluating a culturally adapted behavioral intervention and/or nicotine replacement therapy in Bangladesh, India, and Pakistan. This article presents the process evaluation.

**Methods:**

Mixed methods consisted of interviews with 46 participants and five cessation advisors, a questionnaire completed by 236 trial participants, fidelity assessment of intervention delivery for 38 participants and intervention logs. Data were triangulated across three process evaluation functions (implementation, mechanisms of impact, context) and self-reported abstinence outcomes.

**Results:**

After everyone attending the pre-quit behavioral session, attendance dropped to 86.3% (quit session) and 65.9% (>1 post-quit sessions). Abstainers attended more sessions. Advisors were confident in delivering the intervention, favoring face-to-face over remote, yet fidelity scores showed room for improvement. Attendance and fidelity scores were consistently best in Bangladesh. There was high acceptability and perceived usefulness of the behavioral intervention with an important role for the advisor especially among abstainers. Two-thirds perceived nicotine replacement therapy as useful, higher among abstainers. Taste and side effects were barriers; adherence was highest in India. Perceived drivers to cessation were new knowledge leading to positive attitudes, beliefs in capability to quit, and family support. Perceived barriers were nicotine addiction, social pressure, and easy access to smokeless tobacco.

**Conclusions:**

This process evaluation affirms the feasibility and acceptability of implementing the Behavioral Intervention to support *S*mokeless Tobacco *C*essation in *A*dults and nicotine replacement therapy interventions while identifying important areas for improvement prior to a full effectiveness trial.

**Implications:**

This study provides detailed insights on the feasibility and acceptability of a culturally adapted behavioral intervention and of nicotine replacement therapy for smokeless tobacco cessation in Bangladesh, India, and Pakistan. They identify important implications for the design and delivery of future effectiveness trials of smokeless tobacco cessation interventions that are lacking in this region.

## Introduction

Smokeless tobacco (ST) refers to non-combustible tobacco that is kept in the mouth, chewed, or inhaled nasally.^1^ Consumed by 350 million users worldwide,^2^ ST use is mainly concentrated in South Asia, where it is culturally ingrained and socially acceptable.^3^ In these settings, a range of ST products are available, and are consumed more commonly among females in Bangladesh and among males in India and Pakistan.^4^ ST use is linked to oral and oropharyngeal cancers,^5^^,^^6^ cardiovascular disease,^7^ low birth weight and stillbirth.^8^ These health consequences stem from the toxic and addictive constituents of ST, which contribute to approximately two-thirds of the global burden of ST-related diseases.^2^^,^^9^

The World Health Organization’s (WHO) Framework Convention for Tobacco Control (FCTC) requires member jurisdictions to implement evidence-based measures to reduce tobacco use in all forms.^10^ However, efforts to address ST use lag behind those on smoking, even in high-burden regions like South Asia.^11^^,^^12^ Support toward quitting ST remains weak in South Asian settings-only India has national guidelines and services,^13^ while provision is absent or restricted to a handful of private healthcare facilities in Bangladesh and Pakistan.^11^^,^^14^

Trials conducted in western countries show promise for behavioral and pharmacological interventions for ST cessation.^15^^,^^16^ Three trials were conducted in South Asia in the past decade,^16^ however, none included a process evaluation which could provide insights into the design and delivery of these interventions. Addressing this research gap can enhance the effectiveness and scalability of ST cessation programs in South Asia.

From December 2019 to November 2021, we tested a theory-based behavioral intervention (Behavioral Intervention to support *S*mokeless Tobacco *C*essation in *A*dults—BISCA) alongside nicotine replacement therapy (NRT) in a factorial-design pilot randomized controlled trial.^17^ BISCA is tailored to South Asian ST users,^18^ tested in UK and Pakistani populations,^18^ and adapted for delivery in South Asia.^19^ The BISCA intervention pack comprises an advisor flipbook, a client booklet, and a self-help calendar for ST users. The intervention was delivered face-to-face (and remotely during COVID-19) by cessation advisors who had completed a three day in-person training led by a master trainer. BISCA activities were organized into (1) one pre-quit session focusing on knowledge, self-efficacy and quit preparation (2) at least one quit session, focusing on an ex-user identity, and the identification and management of triggers, and (3) up to six post-quit sessions, focusing on managing withdrawal and preventing relapse.

The pilot trial randomized 264 participants from Bangladesh, India, and Pakistan to BISCA, and/or NRT (4-6 mg nicotine chewing gum for 8 weeks). Participants receiving no active intervention received Very Brief Advice (not reported in this paper). Self-reported continuous abstinence from tobacco (primary outcome) was assessed and biochemically verified (using salivary cotinine) at 26 weeks. Findings indicated a potential benefit of both interventions compared to not receiving the intervention, with higher preliminary effect estimates obtained for BISCA (RR: 2.32, 95% CI = 1.018% to 5.37%) than NRT (RR: 1.25, 95% CI = 0.59% to 2.65%).^20^

In pilot trials, process evaluation is important to understand the feasibility and acceptability of an intervention, as well as optimizing its content and delivery^21^ for future testing. This article reports the findings of the process evaluation of BISCA and NRT that considered three process evaluation functions (implementation, mechanisms of impact, context).^21^

## Materials and Methods

### Overview of Study Design

**Figure 1:**
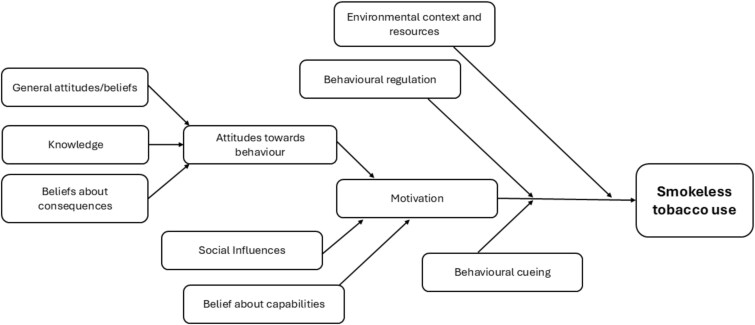
Proposed causal model.

A mixed-methods process evaluation was conducted throughout the trial with activities halted March–June 2020 due to COVID-19. Our design comprised interviews and questionnaires with trial participants, interviews with cessation advisors, fidelity assessment of intervention delivery, and intervention logs. Mechanisms of Action (MoAs)^22^ provided the theoretical underpinning. Datasets were triangulated using meta-themes^23^ reflecting the above mentioned process evaluation functions^21^:

- Implementation—what is delivered (session attendance, fidelity)
- Mechanisms of impact—how does the intervention produce change? (intervention acceptability, MoAs relating to the individual-knowledge, general attitudes/beliefs, beliefs about consequences/capabilities, behavioral cueing/regulation, see Figure 1).^22^
- Context—how does context affect implementation and outcomes? (MoAs relating to the social and physical environment—social influences, environmental context and resources, see Figure 1).^22^

### Interviews

#### Participants

46 trial participants were interviewed (see Table 1), more males in India (80%) and Pakistan (100%), and more females (81%) in Bangladesh, reflecting the gender profile of ST users.^4^ Due to COVID-19 disruption, only five participants were interviewed in Pakistan. We achieved a good mix of participants who did or did not complete the NRT therapy. However, participants receiving BISCA were predominantly those who completed the program. It was difficult to recruit interview participants who had dropped out (only three, all from India).

**Table 1:** Characteristics of Interview Participants

Participant characteristics		Bangladesh (*n* = 21)	India (*n* = 20)	Pakistan (*n* = 5)	All (*n* = 46)
Intervention arm	NRT	4	3	4	11
	BISCA	8	8	1	17
	BISCA + NRT	9	9	0	18
Sex	Male	4	16	5	25
	Female	17	4	0	21
BISCA Programme status	Completed^a^/still doing BISCA sessions	17	14	1	32
	Dropped out from BISCA sessions	0	3	0	3
NRT therapy status	Completed/still taking NRT therapy	7	2	3	12
	Did not complete NRT therapy	6	10	1	17

^a^Completing all BISCA sessions was defined as attending pre-quit session(s), quit session and at least one post-quit session.

All the cessation advisors were interviewed (2 females in Bangladesh, 1 male in India, 1 male and 1 female in Pakistan).

#### Data Collection

Semi-structured interviews with participants were conducted at 6-week follow-up (during the post-quit period of the BISCA intervention) via telephone or face-to-face. Cessation advisors were interviewed face-to-face once intervention delivery was complete. All participants provided informed consent. Interviews with participants explored views of BISCA and/or NRT, perceived impact on quit attempts, and individual or environmental MoAs of behavior change (see Figure 1). These lasted 20–50 min. Interviews with advisors explored their experience of delivery, lasting 35–140 min. All interviews were audio-recorded.

#### Data Analysis

The interviews were transcribed verbatim, checked against audio recordings, then translated into English. Thematic analysis was conducted using the Framework approach,^24^ facilitated by Excel 365 software.

Four English-language thematic frameworks (three trial arms, cessation advisor) were developed based on the topic guides, then piloted with the first interview transcripts, before finalizing. The data (verbatim quotes) were charted into the relevant frameworks. The charted datasets were reviewed to compare views and seek patterns within the data. Descriptive findings documents were written in each country.

### Questionnaire

#### Participants and Data Collection

All participants (*n* = 198) receiving an intervention completed a short process evaluation questionnaire via telephone or face-to-face at 6-week follow-up. It asked questions on acceptability (poor/fair/good/excellent) and perceived usefulness (1-not at all useful to 7-extremely useful).

#### Data Analysis

Acceptability and perceived usefulness were analyzed using frequencies and proportions by country and combined across countries, and cross-tabulated with self-reported abstinence since the quit date at 26 weeks.

### Fidelity Assessment

#### Data Collection

Audio recordings of BISCA sessions for a sub-sample of participants (*n* = 16 Bangladesh, *n* = 6 India, *n* = 16 Pakistan) were purposively selected to ensure all advisors were assessed. Fewer numbers in India reflected the smaller trial sample size.^20^ Two researchers in each country listened to the recordings and completed a fidelity index developed specifically for the BISCA intervention, which comprised two indices (Supplementary File 1).

The *adherence* index assessed adherence to the content of BISCA, BISCA+NRT, and NRT-only. Index items pertained to session ingredients that were mapped to behavior change techniques,^25^ 13, six, and three items for pre-quit, quit, and post-quit BISCA sessions, respectively, and one item for NRT that changed across sessions. The *quality of interaction* index assessed the style of BISCA delivery using seven items applied in the original feasibility testing of BISCA.^18^ Both indices were scored on a three-point Likert scale (0 = not implemented, 1 = partially implemented, 2 = fully implemented). Researchers completed the index independently and then met to agree on the final scores.

#### Data Analysis

The agreed scores for each session ingredient were totaled to provide an *adherence* score for BISCA and NRT sessions and an *interaction* score for BISCA sessions. For each session, means and standard deviations were calculated for by country and combined across countries.

### Intervention Logs

#### Data Collection

Cessation advisors recorded attendance for BISCA sessions and participants’ NRT self-reported adherence in follow-up visits. Face-to-face attendance (versus remote) was recorded in Bangladesh and India, but not in Pakistan.

#### Data Analysis

Counts and percentages were calculated for all items, by country and combined across countries. Mean number of BISCA and NRT sessions attended were analyzed by self-reported continuous abstinence recorded at 26 weeks (for Bangladesh and India only, missing data for Pakistan).

### Triangulating Findings

Key findings for each intervention (BISCA, NRT, BISCA+NRT) were triangulated, structured by the three meta-themes^23^: implementation, mechanisms of impact, and context^21^ and self-reported abstinence status. For each meta-theme, one or more datasets provided findings.

## Findings

The findings are organized by four key findings that crosscut the meta-themes and are supported with Illustrative quotes**.** Differences by country are described where they were observed.

### Finding 1: Attendance to Cessation Sessions Decreased over Time and Varied by Country

Intervention logs showed that all participants receiving NRT attended their designated session. For those receiving BISCA or BISCA+NRT, there was 100% attendance to one pre-quit session, with 12.1% (*n* = 11 Bangladesh, *n* = 5 India, *n* = 0 Pakistan) receiving additional sessions.^20^ Advisors and interview participants attending additional sessions indicated that these were scheduled when participants either lacked the motivation to set a quit date or failed to quit on that date. Quit session attendance was 86.3%, dropping to 65.9% attending one or more post-quit sessions.^20^ The advisors suggested that missed BISCA sessions or drop-out were typically due to losing interest in quitting, personal or family issues, or moving home. The three interview participants who discontinued BISCA were unclear about their reasons.

Self-reported abstainers from Bangladesh and India attended more BISCA sessions than non-abstainers (Mean 5.71, SD 2.84, *n* = 25 versus Mean 4.32, SD 2.99, *n* = 68, *p*<.01). These data were missing in Pakistan.

**Figure 2:**
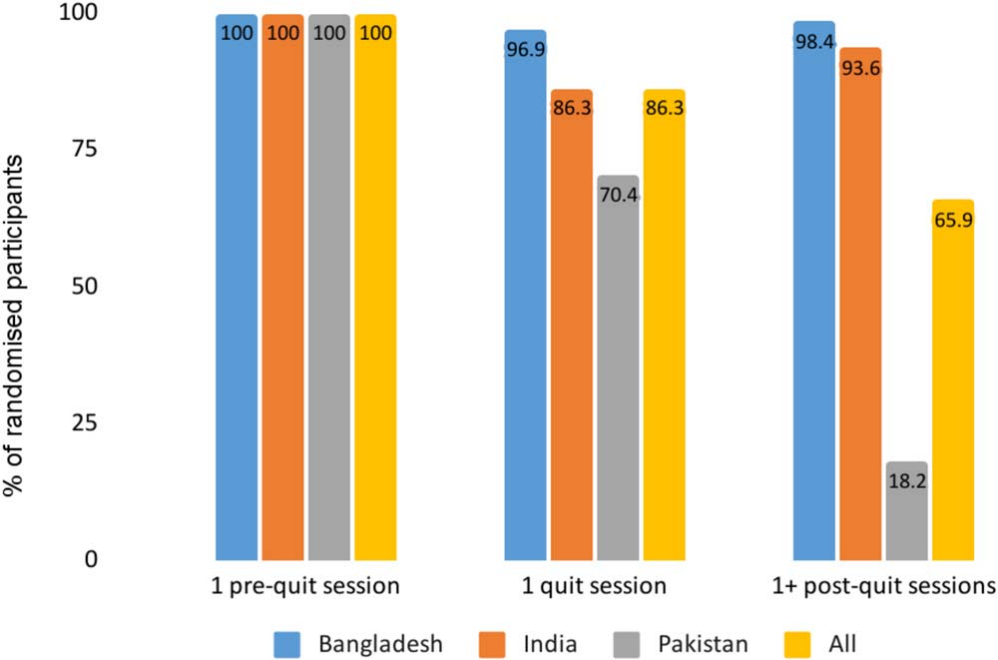
BISCA attendance (%) by session and country.

Attendance was variable between countries across all sessions, highest in Bangladesh and lowest in Pakistan (see Figure 2). The average number of post-quit sessions per participant also differed (Mean 4.8, SD 1.6, *n* = 64 Bangladesh; Mean 1.59, SD 1.33, *n* = 19 India; Mean 0.25, SD 0.61, *n* = 31 Pakistan).^20^

### Finding 2: Cessation Advisors Were Confident in their BISCA and NRT Delivery, but Fidelity Data Showed Room for Improvement with Country Variation

Mixed levels of delivering BISCA were evident across sessions and countries (see Table 2).

**Table 2:** Mean (SD) Fidelity to Delivery of BISCA and NRT Interventions by Country

		Bangladesh		India		Pakistan		All	
	BISCA								
Session	Observed component	*n*	Mean score (SD)	*n*	Mean score (SD)	*n*	Mean score (SD)	*n*	Mean score (SD)
Pre-quit	Adherence	16	21.44 (1.75)	6	18.33 (1.37)	16	17.06 (3.70)	38	19.11 (3.36)
	Interaction	16	11.50 (1.37)	5	10.00 (2.12)	16	10.13 (1.89)	37	10.70 (1.81)
Quit	Adherence	16	10.19 (1.11)	4	5.25 (3.30)	7	8.57 (2.51)	27	9.04 (2.55)
	Interaction	16	11.31 (1.82)	4	10.75 (1.71)	7	9.57 (2.37)	27	10.78 (2.03)
Post-quit 1	Adherence	15	3.80 (0.78)	2	5.00 (1.41)	4	3.29 (1.13)	24	3.75 (0.99)
Post-quit 2	Adherence	15	3.80 (1.21)	0	No data	4	4.00 (1.16)	19	3.84 (1.68)
Post-quit combined	Interaction	16	11.31 (1.74)	0	No data	7	8.71 (3.25)	23	10.52 (2.54)
	**NRT**								
Pre-quit	Adherence	16	1.44 (0.51)	7	1.43 (0.98)	16	1.31 (0.60)	39	1.39 (0.63)
Quit	Adherence	8	1.88 (0.35)	7	1.57 (0.79)	3	1.33 (1.16)	18	1.67 (0.69)
Post-quit 1	Adherence	7	1.43 (0.69)	2	2.00 (00)	3	1.00 (1.00)	12	1.25 (0.75)
Post-quit 2	Adherence	8	1.38 (0.74)	0	No data	3	1.00 (1.41)	10	1.30 (0.82)

Maximum scores: Pre-quit session = 26, quit session = 12, post-quit session = 6, interaction = 14, NRT session = 2.

Mean *adherence* (BISCA content) scores for the pre-quit, quit, and first two post-quit sessions were 19.11 (maximum 26), 9.04 (maximum 12), and 3.75, 3.84 (maximum 6), respectively. Scores were best in Bangladesh for pre-quit and quit sessions (see Table 2). Very little post-quit data were available for India and Pakistan due to the low levels of attendance, precluding comparison.

There were no clear patterns in the fidelity data to indicate well or poorly delivered activities (data available on request) nor did any advisors offer this insight. They said they had delivered all activities as per the BISCA manual. Their only observation regarding the delivery of BISCA content was that face-to-face was easier than telephone delivery because telephone sessions were shorter, and some participants struggled to engage remotely.

…it was not possible on the phone. Firstly, we couldn't see them. Secondly, it was very difficult to explain the calendar to them. (Cessation Advisor, India)

Mean interaction (BISCA delivery) scores were 10.70, 10.78, and 10.52 (maximum 14) across pre-quit, quit, and combined post-quit sessions, with Bangladesh scoring highest (see Table 2). Advisors in all countries, across all sessions scored lowest on the item “Prompt questions from the client and answer clearly and accurately” (data available on request).

Mean adherence (content) scores for the pre-quit, quit, and first two post-quit NRT sessions were 1.39, 1.67, 1.25, and 1.30 (maximum 2), respectively. These were similar across (see Table 2), but limited post-quit data in India and Pakistan precluded comparison. Researcher observations in the fidelity tool indicated that advisors were down-scored for failing to provide “full explanations,” eg, what is NRT, or not asking for sufficient detail on participants’ ST chewing routines. Advisors readily described their NRT conversations as easy to deliver.

### Finding 3: BISCA Was Highly Acceptable and Perceived Useful, with an Important Role for the Cessation Advisor among those Reporting Abstinence. Feedback on NRT Was Mixed with Variation by Country and Abstinence Status

The venue and session times were highly acceptable across countries with 99.5% and 100% of questionnaire respondents rating good or excellent respectively, irrespective of abstinence status (Table S1A–D). Interview participants liked jointly arranging sessions with advisors to fit their routine schedules. Some advisors found arranging remote sessions more challenging. They also offered feedback on BISCA session duration, citing 45–50 min for an average pre-quit session, with more time needed for older, less educated, or less motivated participants. Quit and post-quit sessions were typically 15–25 min across countries, which advisors perceived as sufficient.

Interview participants spoke positively about BISCA. Popular activities were learning about the health-related harms of ST, discussing alternatives to ST consumption and acknowledging the benefits of quitting for their family, especially saving money.

One thing is that after quitting this [Pan], my money has been saved. Every day, I needed 20 Taka extra for this [Pan]. Now, I do not need this extra money. (Female, Bangladesh, BISCA+NRT arm)

Advisors were described as supportive, experienced, and good communicators. Counseling and setting a quit date were seen as motivational; almost all interview participants were determined to quit ST at this time. The questionnaire data confirmed the importance of the advisor, with all respondents rating them good or excellent. Overall, 80.0% strongly agreed that interactions with an advisor helped their quit attempt, more so among abstainers (90.1% versus 63.0%, Tables S1E–H)

The advisor was really knowledgeable. They talked and explained things very well. Sometimes, an explanation can change someone’s mind. (Female, Bangladesh, BISCA+NRT arm)

BISCA resources were also highly acceptable across all countries. The flipbook, self-help calendar and client booklet were rated good or excellent by 96.7%, 95.9%, and 95.9%, respectively. There were no differences for BISCA resources by self-reported abstinence, except for the client booklet, which received higher “excellent” ratings from abstainers than non-abstainers (46.8% versus 38.4%, Tables S2A–F). Advisors suggested using less text and more pictures for participants with low literacy, and avoiding repetition of content.

I think that some people do not have patience for all the reading, and some cannot read. But the pictures and photos we showed stayed in their mind for a long time. If we could add more pictures, it would bring better results. (Advisor, Bangladesh)

Acceptability of NRT was mixed. 83.1% rated it as good or excellent. Two-thirds (65.1%) agreed or strongly agreed that NRT was useful in their quit attempt; higher strongly agree ratings were reported by abstainers (52.9.% v 26.9%, Tables S3A–D). However, self-reported adherence was just 54.5% at 6 weeks with considerable variation across countries (45.5% Bangladesh, 90.9% India, 50% Pakistan).

Interview participants who completed NRT mostly found it easy to take with no complaints about side effects or taste, likening it to tobacco, regular chewing gum, or mint. Of note, intervention logs indicated that more brands and flavors were offered in India.

The taste [of the gum] feels strong in the mouth. I put it in my mouth, the way I would consume tobacco, near the teeth. I kept it for half an hour in the mouth. If I chew it two to four times, I get the same taste as tobacco. (Female, India, NRT arm)

Those who did not complete NRT took it for a few days up to 3 weeks. Some participants in Bangladesh and Pakistan discussed problems with forgetfulness, and difficulty in carrying the gum outside the home. However, the taste (described as bitter, peppery, or extra-sweet) and side effects were the main reason for stopping. Participants described feeling dizzy, sleepy, uneasy, nauseous, having headaches, chest pain, vomiting, cracks on the tongue, and perceived damage to oral mucosa and teeth.

She gave me chewing gum, but I could not use it because I felt like vomiting. I felt pain in my chest, and I vomited once. That is why I did not continue. After eating the first day, I vomited a lot, I felt uneasy. (Female, Bangladesh, NRT arm)

The advisors in Bangladesh and Pakistan attributed low NRT adherence to side effects.

They initially took the NRT very gladly, but many could not use it because of side effects, especially dizziness more so in females. Their head spun and their vision became foggy. We initially suggested NRT for two weeks, but they often could not finish the first dose and returned it to us. (Advisor, Bangladesh)

In Bangladesh they further hypothesized that NRT is new; people are not familiar with chewing gum hourly (up to 15 doses per day) and see it as “irritating.” This perspective was different from that of the advisors in India who believed that most had benefited from it both physically and psychologically (instilling confidence to quit).

### Finding 4: Perceived Drivers to Cessation Were New Knowledge Leading to Positive Attitudes and Beliefs in the Capability to Quit, alongside Family Support. Perceived Barriers Were Nicotine Addiction, Social Pressure, and Easy Access to ST

Interview participants described gaining knowledge that had changed their attitudes, beliefs about consequences, and their capability to quit ST. Family members were mentioned as important sources of motivation and support, with examples of attending cessation sessions, praise for quitting, and disapproval of ST use.

My family members used to go with me [to the cessation sessions]. Before, they were very upset with my oral tobacco powder addiction. They made me throw away so many boxes of tobacco; I used to [verbally] abuse them and argue with them. (Male, India, BISCA arm)

Conversely, strong addiction to ST was seen as the main barrier to quitting, hindering behavioral regulation. Urges and cravings after meals were common, and withdrawal symptoms included chest heaviness, irritability, vertigo, and nausea.

In a day, how should I tell you…my tobacco consumption was about 15-20 times in a day. I consumed so much oral tobacco powder. You understand? My addiction was very bad. (Female, India, NRT arm)

The advisors recognized the need for strong motivation to overcome addiction. They believed they had increased the knowledge and confidence of participants but were realistic about how many had sufficient and enduring motivation to quit.

I felt that many [participants] didn't have that much motivation where they could bear the adverse effects [of quitting ST] because they could not concentrate on their work. So they have the knowledge because they have been given this in the intervention, but I would say addiction has played an important part. (Advisor, India)

Spending time with others who use ST was a trigger and many participants described now avoiding those people or doing a replacement activity in their company.

Many people are addicted to tobacco, but I stay away from such people now. At work when talking to others who use chewable tobacco, they often offered it to me so it was difficult to stop. Now, if I see someone eating Gutka at work, I don't care, I don't sit among these people anymore. (Male, Pakistan, BISCA+NRT arm)

The advisors spoke of how readily available ST is, both in terms of access and cost, seeing it as a behavioral cue to ST use. Conversely, a few participants in India reported a lack of ST products in markets during the pandemic.

## Discussion

The process evaluation of this multi-country pilot trial revealed that BISCA and advisors are highly acceptable. Participants reported increased knowledge, motivation, and confidence to quit ST. There is room for improving delivery and attendance to quit and post-quit sessions in India and Pakistan. Abstainers attended more BISCA sessions than non-abstainers. Adherence to, and acceptability of, NRT was best in India. Abstainers rated the advisor, client booklet, and usefulness of NRT more highly than non-abstainers. This knowledge, together with data on study feasibility and preliminary data on ST cessation,^20^ have been used to inform the design of a smoking and ST cessation intervention to be evaluated in a trial in Bangladesh, India, and Pakistan (https://www.impactsouthasia.com/scimitar-sa/).

The BISCA intervention and its resources were rated as highly acceptable by participants. However, advisors highlighted the need to improve content accessibility by reducing text and including more visual cues. Similar findings emerged from a process evaluation of behavioral activation delivered in South Asian settings,^26^ and have particular relevance for ST given its use among those with low levels of education.^4^ Abstainers attended more BISCA sessions than non-abstainers, there was limited use of additional pre-quit sessions, and poor attendance in post-quit sessions, particularly in India and Pakistan. Multiple factors might have influenced attendance, including perceived benefit, support from family and friends, accessibility, and convenience.^27^ Informal conversations with researchers suggested that more support was provided for scheduling sessions in Bangladesh. Participants rated advisors favorably, especially those who abstained from ST.

Fidelity assessments highlighted room for improvement in both the content and interaction aspects of BISCA and NRT delivery, especially in India and Pakistan. In the future, advisor training should lay emphasis on closer adherence to content delivery, advisor-participant interaction, and person-centered communication. These strategies can effectively improve client engagement and satisfaction, increasing the potential for quit success.^28^

NRT has proven effectiveness in treating tobacco dependence, particularly alongside behavioral interventions,^29^ and ensuring adherence can predict future abstinence.^30^ Participants generally perceived NRT as acceptable, but fewer saw it as useful to their quit attempt. Adherence was 90.9% in India, yet only moderate levels were observed in Bangladesh and Pakistan, consistent with previous research.^31^ This difference may be due to NRT being more established as a cessation aid in India with a wider range of products, or greater emphasis on NRT by these advisors given the Master trainer’s significant experience of NRT. We tailored NRT dosing and frequency to participants' dependence levels which has shown to result in successful quit attempts.^32^ However, our findings suggest the need to enhance adherence. It is important to note here that NRT was only provided in gum form. Increased optionality (eg, nicotine lozenges or nasal sprays) could potentially increase its uptake among those who prefer non-chewing options, either due to personal or social reasons. Also, the provision of additional information and support, particularly around side effects of NRT, and their management should be considered, as similar strategies have been shown to improve knowledge and adherence elsewhere.^33^^,^^34^ The provision of alternate NRT flavors, as was done in India, and other pharmacotherapies (eg, varenicline and cytisine) should also be explored.^35^^,^^36^

This pilot trial faced challenging situations, notably the displacement of participants from slum dwellings in Dhaka and COVID-19 lockdowns across all countries. Moving the intervention to a hybrid/remote format in response to these challenges created scheduling and communication challenges for advisors. In the UK, stop smoking practitioners faced similar challenges during the pandemic, but in contrast to our findings, reported improved attendance and engagement among clients.^37^ A possible reason could be the differences in support available to advisors. To achieve similar outcomes, it is thus imperative to bolster administrative, logistic, and training support for remote delivery. Face-to-face delivery should be retained as the first choice, given participants’ positive reviews and advisors' preferences in our study, alongside mixed evidence on the comparability of remote and face to face sessions.^38^^,^^39^

### Strengths and Limitations

This process evaluation comprised four data sets triangulated to understand three process evaluation functions. This approach, based on good practice,^21^^,^^23^ gives us confidence in the findings. By using the same approach across countries, we identified differences and shared lessons of good practice. There were, however, some gaps in the data. We have no formal records on the delivery format of BISCA sessions (face-to-face or remote), only a perception from research teams that despite the pandemic, “most” sessions were delivered face-to-face. This precludes us from understanding if drop-off in BISCA attendance could be alleviated by changing the delivery format. We also have no interview data on reasons for BISCA drop-out. Finally, we had fewer interview participants in Pakistan limiting the in-depth feedback we could collect. The imbalance in the gender profile of interview participants is less of a concern as it mirrors ST users in each country.^4^

## Conclusions

This process evaluation found that both BISCA and NRT are feasible and acceptable, but their uptake was variable across countries. To improve participant engagement and outcomes, BISCA materials need to be made accessible to individuals with lower levels of literacy. Enhanced training and support for cessation advisors is also needed, particularly on some features of BISCA and NRT content, advisor-participant interaction and remote delivery. These findings have important implications for the design and delivery of future effectiveness trials that deliver behavioral interventions and/or pharmacotherapy for ST cessation in this region.

## Supplementary Material

Supplementary_File_1-_Fidelity_Index_ntag004

Supplementary_File_2_-_General_FINAL_ntag004

Supplementary_File_3-_BISCA_FINAL_ntag004

Supplementary_File_4_-_NRT_FINAL_ntag004

## Data Availability

The data that support the findings of this study are available from the corresponding author upon reasonable request.

## Notes

Fariza Fieroze is now at Independent Researcher

Laraib Mazhar is now at Penn State College of Medicine
