# Supplementary material for: Mixed Methods Process Evaluation of Behavioral Support and Nicotine Replacement Therapy for Smokeless Tobacco Cessation in Bangladesh, India, and Pakistan
Source: Nicotine Tob Res. 2026 Jan 8;28(6):956–64. doi: 10.1093/ntr/ntag004 (PMC13196702; doi:10.1093/ntr/ntag004)
Supplement: Supplementary_File_2_-_General_FINAL_ntag004 [file supplementary_file_2_-_general_final_ntag004.docx]

**Supplementary File 1: General Feedback**

Supplementary Table 1A: Acceptability of the venue

| Trial arm | Rating | | | Total |
| --- | --- | --- | --- | --- |
|  | Fair | Good | Excellent |  |
| BISCA | 1 | 30 | 29 | 60 |
| BISCA+NRT | 0 | 33 | 30 | 63 |
| NRT | 0 | 41 | 22 | 63 |
| Total | 1 | 104 | 81 | 186 |

Supplementary Table 1B: Acceptability of the venue by self-reported abstinence at 26 weeks

|  | Rating | | | Total |
| --- | --- | --- | --- | --- |
|  | Fair | Good | Excellent |  |
| Abstained | 1 | 61 | 44 | 106 |
| Not abstained | 0 | 42 | 31 | 73 |
| Total | 1 | 103 | 74 | 179 |

Supplementary Table 1C: Acceptability of the time of appointments

| Trial arm | Rating | | Total |
| --- | --- | --- | --- |
|  | Good | Excellent |  |
| BISCA | 29 | 31 | 60 |
| BISCA+NRT | 25 | 38 | 63 |
| NRT | 34 | 29 | 63 |
| Total | 88 | 98 | 186 |

Supplementary Table 1D: Acceptability of the time of appointments by self-reported abstinence at 26 weeks

|  | Rating | | Total |
| --- | --- | --- | --- |
|  | Good | Excellent |  |
| Abstained | 50 | 56 | 106 |
| Not abstained | 38 | 35 | 73 |
| Total | 88 | 91 | 179 |

Supplementary Table 1E: Acceptability of the advisor

| Trial arm | Rating | | Total |
| --- | --- | --- | --- |
|  | Good | Excellent |  |
| BISCA | 10 | 50 | 60 |
| BISCA+NRT | 7 | 56 | 63 |
| NRT | 17 | 46 | 63 |
| Total | 34 | 152 | 186 |

Supplementary Table 1F: Acceptability of the advisor by self-reported abstinence at 26 weeks

|  | Rating | | Total |
| --- | --- | --- | --- |
|  | Good | Excellent |  |
| Abstained | 18 | 88 | 106 |
| Not abstained | 16 | 57 | 73 |
| Total | 34 | 145 | 179 |

Supplementary Table 1G: Perceived usefulness of the advisor

|  | The conversations I had with my advisor were useful in supporting my quit attempt | | | | | Total |
| --- | --- | --- | --- | --- | --- | --- |
|  | Strongly agree | Agree | Neither | Disagree | Strongly disagree |  |
| BISCA | 48 | 6 | 3 | 2 | 1 | 60 |
| BISCA+NRT | 56 | 4 | 3 | 0 | 0 | 63 |
| NRT | 45 | 6 | 12 | 0 | 0 | 63 |
| Total | 149 | 16 | 18 | 2 | 1 | 186 |

Supplementary Table 1H: Perceived usefulness of the advisor by self-reported abstinence at 26 weeks

|  | The conversations I had with my advisor were useful in supporting my quit attempt | | | | | Total |
| --- | --- | --- | --- | --- | --- | --- |
|  | Strongly agree | Agree | Neither | Disagree | Strongly disagree |  |
| Abstained | 96 | 7 | 2 | 0 | 1 | 106 |
| Not abstained | 46 | 9 | 16 | 2 | 0 | 73 |
| Total | 142 | 16 | 18 | 2 | 1 | 179 |
