# Supplementary material for: Mixed Methods Process Evaluation of Behavioral Support and Nicotine Replacement Therapy for Smokeless Tobacco Cessation in Bangladesh, India, and Pakistan
Source: Nicotine Tob Res. 2026 Jan 8;28(6):956–64. doi: 10.1093/ntr/ntag004 (PMC13196702; doi:10.1093/ntr/ntag004)
Supplement: Supplementary_File_3-_BISCA_FINAL_ntag004 [file supplementary_file_3-_bisca_final_ntag004.docx]

**Supplementary File 2: BISCA Feedback**

Supplementary Table 2A: Acceptability of the flipbook

| Trial arm | Rating | | | Total |
| --- | --- | --- | --- | --- |
|  | Did not use it | Good | Excellent |  |
| BISCA | 1 | 34 | 25 | 60 |
| BISCA+NRT | 3 | 33 | 27 | 63 |
| Total | 4 | 67 | 52 | 123 |

Supplementary Table 2B: Acceptability of the flipbook by self-reported abstinence at 26 weeks

|  | Rating | | | Total |
| --- | --- | --- | --- | --- |
|  | Did not use it | Good | Excellent |  |
| Abstained | 2 | 46 | 31 | 79 |
| Not abstained | 2 | 21 | 16 | 39 |
| Total | 4 | 47 | 67 | 118 |

Supplementary Table 2C: Acceptability of the calendar

| Trial arm | Rating | | | Total |
| --- | --- | --- | --- | --- |
|  | Did not use it | Good | Excellent |  |
| BISCA | 2 | 29 | 29 | 60 |
| BISCA+NRT | 3 | 26 | 34 | 63 |
| Total | 5 | 55 | 63 | 123 |

Supplementary Table 2D: Acceptability of the calendar by self-reported abstinence at 26 weeks

|  | Rating | | | Total |
| --- | --- | --- | --- | --- |
|  | Did not use it | Good | Excellent |  |
| Abstained | 3 | 36 | 40 | 79 |
| Not abstained | 2 | 19 | 18 | 39 |
| Total | 5 | 55 | 58 | 118 |

Supplementary Table 2E: Acceptability of the client booklet

| Trial arm | Rating | | | Total |
| --- | --- | --- | --- | --- |
|  | Did not use it | Good | Excellent |  |
| BISCA | 2 | 32 | 26 | 60 |
| BISCA+NRT | 3 | 29 | 31 | 63 |
| Total | 5 | 61 | 57 | 123 |

Supplementary Table 2F: Acceptability of the client booklet by self-reported abstinence at 26 weeks

|  | Rating | | | Total |
| --- | --- | --- | --- | --- |
|  | Did not use it | Good | Excellent |  |
| Abstained | 3 | 39 | 37 | 79 |
| Not abstained | 2 | 22 | 15 | 39 |
| Total | 5 | 61 | 52 | 118 |
