# Supplementary material for: Mixed Methods Process Evaluation of Behavioral Support and Nicotine Replacement Therapy for Smokeless Tobacco Cessation in Bangladesh, India, and Pakistan
Source: Nicotine Tob Res. 2026 Jan 8;28(6):956–64. doi: 10.1093/ntr/ntag004 (PMC13196702; doi:10.1093/ntr/ntag004)
Supplement: Supplementary_File_4_-_NRT_FINAL_ntag004 [file supplementary_file_4_-_nrt_final_ntag004.docx]

**Supplementary File 3: NRT Feedback**

Supplementary Table 3A: Acceptability of NRT

| Trial arm | Rating | | | | | Total |
| --- | --- | --- | --- | --- | --- | --- |
|  | Did not use it | Poor | Fair | Good | Excellent |  |
| NRT | 0 | 2 | 9 | 37 | 13 | 61 |
| BISCA+NRT | 1 | 3 | 6 | 33 | 20 | 63 |
| Total | 1 | 5 | 15 | 70 | 33 | 124 |

Supplementary Table 3B: Acceptability of NRT by self-reported abstinence at 26 weeks

|  | Rating | | | | | Total |
| --- | --- | --- | --- | --- | --- | --- |
|  | Did not use it | Poor | Fair | Good | Excellent |  |
| Abstained | 0 | 1 | 5 | 37 | 25 | 68 |
| Not abstained | 1 | 4 | 7 | 30 | 8 | 50 |
| Total | 1 | 5 | 12 | 67 | 33 | 118 |

Supplementary Table 3C: Perceived usefulness of NRT

|  | NRT was useful in supporting my quit attempt | | | | | Total |
| --- | --- | --- | --- | --- | --- | --- |
|  | Strongly agree | Agree | Neither | Disagree | Strongly disagree |  |
| BISCA+NRT | 26 | 16 | 16 | 2 | 3 | 63 |
| NRT | 25 | 15 | 21 | 0 | 2 | 63 |
| Total | 51 | 31 | 37 | 2 | 5 | 126 |

Supplementary Table 3D: Perceived usefulness of NRT by self-reported abstinence at 26 weeks

|  | NRT was useful in supporting my quit attempt | | | | | Total |
| --- | --- | --- | --- | --- | --- | --- |
|  | Strongly agree | Agree | Neither | Disagree | Strongly disagree |  |
| Abstained | 36 | 13 | 16 | 2 | 1 | 68 |
| Not abstained | 14 | 16 | 18 | 0 | 4 | 52 |
| Total | 50 | 29 | 34 | 2 | 5 | 120 |
